# Leronlimab, a humanized monoclonal antibody to CCR5, blocks breast cancer cellular metastasis and enhances cell death induced by DNA damaging chemotherapy

**DOI:** 10.1186/s13058-021-01391-1

**Published:** 2021-01-23

**Authors:** Xuanmao Jiao, Min Wang, Zhao Zhang, Zhiping Li, Dong Ni, Anthony W. Ashton, Hsin-Yao Tang, David W. Speicher, Richard G. Pestell

**Affiliations:** 1 Pennsylvania Cancer and Regenerative Medicine Research Center, Baruch S. Blumberg Institute, Pennsylvania Biotechnology Center, 100 East Lancaster Avenue, LIMR R234, Wynnewood, PA 19096 USA; 2 Division of Perinatal Research, Kolling Institute, Northern Sydney Local Health District, St Leonards, NSW 2065 Australia; 3 Sydney Medical School Northern, University of Sydney, Sydney, NSW 2006 Australia; 4 Wistar Institute, Philadelphia, PA 19107 USA; 5 Xavier University School of Medicine, 1000 Woodbury Rd, Suite 109, Woodbury, NY 11797 USA

**Keywords:** Leronlimab, CCR5, Metastasis, Breast cancer

## Abstract

**Background:**

Triple-negative breast cancer (BCa) (TNBC) is a deadly form of human BCa with limited treatment options and poor prognosis. In our prior analysis of over 2200 breast cancer samples, the G protein-coupled receptor CCR5 was expressed in > 95% of TNBC samples. A humanized monoclonal antibody to CCR5 (leronlimab), used in the treatment of HIV-infected patients, has shown minimal side effects in large patient populations.

**Methods:**

A humanized monoclonal antibody to CCR5, leronlimab, was used for the first time in tissue culture and in mice to determine binding characteristics to human breast cancer cells, intracellular signaling, and impact on (i) metastasis prevention and (ii) impact on established metastasis.

**Results:**

Herein, leronlimab was shown to bind CCR5 in multiple breast cancer cell lines. Binding of leronlimab to CCR5 reduced ligand-induced Ca^+ 2^ signaling, invasion of TNBC into Matrigel, and transwell migration. Leronlimab enhanced the BCa cell killing of the BCa chemotherapy reagent, doxorubicin. In xenografts conducted with Nu/Nu mice, leronlimab reduced lung metastasis of the TNBC cell line, MB-MDA-231, by > 98% at 6 weeks. Treatment with leronlimab reduced the metastatic tumor burden of established TNBC lung metastasis.

**Conclusions:**

The safety profile of leronlimab, together with strong preclinical evidence to both prevent and reduce established breast cancer metastasis herein, suggests studies of clinical efficacy may be warranted.

**Supplementary Information:**

The online version contains supplementary material available at 10.1186/s13058-021-01391-1.

## Introduction

Breast cancer (BCa) remains the most common malignancy in women other than skin cancer, representing approximately one third of all malignancies diagnosed among women in the USA [1, 2]. Since 2008, the incidence of breast cancer worldwide has increased by more than 20%, and mortality has increased by 14%. In order to treat breast cancer more precisely, several genetic drivers of breast cancer have been identified, and subclassification has been conducted based on either the coding or non-coding genome. The classification of breast cancer based on the coding region identified potential genetic targets including the *CCND1* gene, which is amplified in 30 to 58% of breast cancers; the estrogen receptor (ERα); and/or progesterone receptor (PR) and Her2. Because evidence suggests both the coding and non-coding genome may contribute to the onset and progression of tumorigenesis [3, 4], subtypes of breast cancer have been identified using patterns of expression for both the coding [5] and non-coding genomes [6–8].

Using the coding genome, five distinct molecular subtypes were identified referred to as luminal A, luminal B, human epidermal growth factor receptor 2 (HER2)-enriched, basal-like, and claudin-low and normal-like [9]. Triple-negative breast cancer (TNBC), which lacks ERα, PR, and Her2, is a deadly form of breast cancer. In 10 to 15% of cases, TNBC is associated with DNA damage repair protein mutations (*BRCA1 BARD1*, *BRCA1*, *BRCA2*, *PALB2*, and *RAD51D)* [10], in 19% with PD-L1 expression [11], and in > 95% with CCR5 overexpression [12].

Well known as an essential co-receptor for HIV, more recently, CCR5 has become strongly implicated in the progression of human cancer, in particular, metastatic cancer [13]. CCR5, a seven trans-membrane G-protein coupled receptor (GPCR), is normally expressed only in the immune system; however, CCR5 becomes overexpressed in several malignancies and is overexpressed in breast cancer [12, 13]. In the analysis of > 2200 breast cancer patients, > 50% of patient’s tumors were CCR5^+.^ and > 95% of triple-negative breast cancer (TNBC) were CCR5^+^ [12]. Several characteristics of CCR5 suggest the receptor may be important in human breast cancer. CCR5 receptor levels correlate with poor prognosis in breast cancer [13–15]. CCR5 expression correlates well with increased tumor heterogeneity in breast cancer [16, 17]. Upon transformation of breast epithelial cells, the increased expression of CCR5 results in increased motility and homing behavior to metastatic sites [12, 13]. Furthermore, CCR5^+^ breast cancer epithelial cells have both enhanced tumor-initiating capacity and form mammospheres with greater efficiency in mice [13], a feature of cancer stem cells. Finally, ectopic CCR5 expression within cancer epithelial cells is sufficient to drive cancer cell metastasis [12].

Several CCR5 antagonists developed for HIV treatment, including the small molecule CCR5 inhibitors (maraviroc and vicriviroc) and the humanized monoclonal anti-CCR5 antibody leronlimab, are currently being retasked for cancer and cancer-related diseases [17, 18]. In HIV treatment, the small-molecule inhibitor maraviroc and the humanized monoclonal antibody leronlimab achieved their primary endpoints in phase 3 HIV clinical trials [19–21]. CCR5-specific small molecular inhibitors prevented metastasis of isogenic oncogene-transformed breast cancer cells in NOD/SCID mice [12] and prostate cancer metastasis in immune-competent mice [22]. Unfortunately, maraviroc carries a “black box” warning due to the associated serious adverse including hepatotoxicity.

Leronlimab is an inhibitor of CCR5 signaling in immune cells. Currently, more than 800 patients with HIV have received leronlimab without serious adverse events related to the agent. Given the safety profile of leronlimab, and potential adverse events with the small molecular inhibitors, we conducted studies to determine whether leronlimab could bind and block CCR5 signaling in human breast cancer cells. These studies extend prior studies by showing CCR5 inhibition both prevents metastasis and reduces the progression of established metastasis in vivo.

## Materials and methods

### Reagents and antibodies

Human CCL3, CCL4, CCL5, and APC conjunct mouse anti-human/mouse/rat CCR5 antibody (FAB1802A) were purchased from R&D Systems. Rat tail collagen type I was purchased from BD Biosciences. Maraviroc, vicriviroc, and luciferin was purchased from Selleck Chemicals. Leronlimab, a fully humanized monoclonal IgG4 antibody that was developed as an entry inhibitor for HIV [23], was provided by CytoDyn Inc. Doxorubicin was obtained from Sigma.

### Cell lines, plasmids, and cell culture

MDA-MB-231 and MDA-MB-231-CCR5 stable cells [12, 22, 24] were maintained in Dulbecco’s modified Eagle’s medium (DMEM) supplemented with 10% FBS, 100 IU/mL penicillin, and 100 μg/ml streptomycin. The CCR5 expression vector which encodes full-length human CCR5 by subcloning into pcDNA3.1^+^/Zeo^+^ vector was kindly provided by Dr. Eleanor Fish at University of Toronto, Toronto, ON, Canada [25], and the cell line was selected with Zeocin (200 μg/mL). The luciferase construct Luc2-eGFP is a lentiviral vector encoding firefly luciferase 2 (Luc2)-eGFP fusion protein and was a generous gift from Dr. Sanjiv S. Gambhir (School of Medicine, Stanford University, Stanford, CA) [26]. Lentivirus propagation was conducted following the protocol described by Zahler and colleagues [27]. Cells were cultured in 5% CO_2_ at 37 °C. For in vitro treatments, maraviroc was dissolved in dimethyl sulfoxide (DMSO) and diluted in a culture medium. The final concentration of DMSO in treated and control cultures was 0.5%. Vicriviroc was dissolved in a culture medium.

### Fluorescence-activated cell-sorting analysis

Cell labeling and fluorescence-activated cell-sorting (FACS) analysis for CCR5 were based on prior publications [13, 28]. Before labeling, the cells were blocked with normal mouse IgG (1/100) for 1 h and then incubated with allophycocyanin (APC)-labeled CCR5 antibody (R&D Systems). All experiments were conducted at 4 °C. FACS sorting was conducted on FACS-Canto flow cytometer (BD Biosciences), and the data were analyzed with the FlowJo software (Tree Star, Inc.).

### Invasion assays

The 3-dimensional transwell invasion assays were conducted as previously reported [12, 22, 24]. Briefly, 100 μL of 1.67 mg/mL Rat tail collagen type I (BD Biosciences) was pipetted into the top chamber of a 24-well 8-mm pore transwell (Corning). The transwell was incubated at 37 °C for more than 30 min allowing the collagen to solidify. The transwell was inverted, and a total of 30,000 cells in 100 μL of serum-free medium were seeded on the bottom of the transwell and then incubated at 37 °C, in a 5% CO_2_ incubator for 4 h to allow the cells to attach to the transwell membrane. Serum-free growth medium was placed into the bottom chamber, whereas 20 ng/mL CCL5 or 5% FBS was added as the chemoattractant in the medium of the upper chamber. The cells were then chemoattracted across the filter through the collagen above for 3 days. Cells were fixed in 10% formalin in PBS and then stained with 40 mg/mL propidium iodide (PI) for 2 h. Fluorescence was analyzed in z-sections mode using × 10 objective lens with Nikon C2+ confocal inverted microscope at the Lankenau Institute for Medical Research Bioimaging Facility.

### Intracellular calcium assay

Calcium responses induced either by ligand (CCL5, CCL3, or CCL4) or FBS in the MDA-MB-231 breast cancer cell line were monitored under a fluorescence microscope as previously reported [12, 29]. Breast cancer cells were seeded in a 4-well Labtech II chamber coverglass (Nunc) at 10^4^ cells/cm^2^ and incubated for 1 day. After 12-h of starvation, cells were labeled by incubating them with 2 μmol/L Fluo-4-AM (Molecular Probes) in HBSS for 30 min, washed once, and incubated for an additional 30 min before imaging under the microscope. Time-lapse images were collected using a Zeiss Axivert 200 M inverted fluorescent microscope with the incubator at 37 °C, 5% CO_2_. Relative intracellular Ca^2+^ concentration was determined by the changes in fluorescent intensity (FI) of Fluo-4-AM upon the addition of the ligands or FBS and was calculated as (*FI*_*t*_ − *FI*_min_)/(*F*_max_ − *FI*_min_).

### MTT assay

The effects of leronlimab and doxorubicin on cell viability and proliferation rate were estimated using the soluble tetrazolium salt MTT assay [30]. MTT is reduced by the mitochondria of viable cells, and the amount of reduced formazan is proportional to the number of viable cells. Equal numbers of cells were plated, and after 72 h of exposure to the drugs, cells were incubated with 1 mg/mL of MTT for 90 min. The reduced (insoluble and colored) formazan was dissolved in 0.04 N HCl acidified isopropanol and measured spectrophotometrically at 570 nm. The MTT assay activity was compared between equimolar amounts of control IgG (human, Sigma, #56834) and leronlimab. The normalized absorbance was calculated by dividing the absorbance values of doxorubicin treated samples in each group with their respective untreated control and was shown as “relative absorbance as a fraction of the untreated control”.

### Experimental metastasis assay and bioluminescence imaging

Animal experiments were approved by the Lankenau Institute for Medical Research Institutional Animal Care and Use Committee (IACUC). MB-MDA-231 cells expressing Luc2-eGFP (called MDA. pFLUG for the rest of the article) were detached with 0.25% trypsin-EDTA, washed twice with PBS and resuspended in PBS with 10^7^ cells/ml, and immediately injected into the tail vein of 8-week-old, female NCI Athymic nu/nu nude mice (Charles River). Each mouse received 10^6^ cells. Mice were treated with leronlimab (intraperitoneal (IP) injection and 2 mg/mouse twice a week). The dose of 2 mg/mouse was calculated to approximate the dose used in the sponsored phase II human clinical trial for acute GVHD [31], or maraviroc by oral gavage (8 mg/kg every twice a day) [12]. Untreated mice were used as control. Treatment was started 1 day before to determine the impact on the prevention of metastasis or 7 weeks after tumor cell injection to determine the impact on established metastasis. For in vivo bioluminescence imaging (BLI), mice were given an intraperitoneal (IP) injection with 100 μL of D-luciferin (30 mg/mL) and anesthetized with isoflurane (2% in 1 L/min oxygen). Bioluminescence images were acquired with the IVIS XR system (Caliper Life Sciences) 10–15 min after D-luciferin injection. Acquisition times ranged from 10 s (for later time points) to 5 min (for early time points). Data are expressed as total photon flux and were analyzed using the Living Image 3.0 software (Caliper Life Sciences).

### Transwell migration assays

Transwell migration assays were performed as described before [32]. Briefly, 8-μm-pore-size Transwell filter insert (Costar) was coated overnight with 0.5% of Matrigel (#356234, Corning, Life Sciences, Tewksbury, MA) in PBS. A total of 10^5^ breast cancer cells in serum-free media were then seeded on the membrane of the Transwell filter insert and incubate for 1 h allowed to attach. A serum-free growth medium with 20 ng/ml of CCL5 used as a chemoattractant was placed into the bottom chamber. The cells were then chemoattracted across the filter. After 7 h of incubation at 37 °C and 5% CO_2_, the cells adherent to the upper surface of the filter were removed using a cotton applicator. The cells on the inverted side of the filter membrane were fixed with 3.7% formaldehyde, stained with crystal violet, and imaged with a microscope. The number of cells in each image were counted by Fiji Image J.

### Histology and quantitation of tumor metastasis

The mice were euthanized, and the lungs were excised. The lungs were immersed in 10% neutral buffered formalin and after fixation for 16 h, and the lungs were placed into 70% ethanol prior to paraffin embedding. Longitudinal sections (4 μm) of the entire lung were obtained every 100 μm. The sections were deparaffinized and stained with hematoxylin and eosin. Each section was evaluated to identify lesions and to differentiate lesions from other space-occupying alterations including consolidation and inflammation. From the 10 sections, section number 5 was digitally imaged, and the region of metastasis was quantified using Fiji ImageJ.

## Results

### The binding of leronlimab with CCR5 expressed in breast cancer cells

**Fig. 1:**
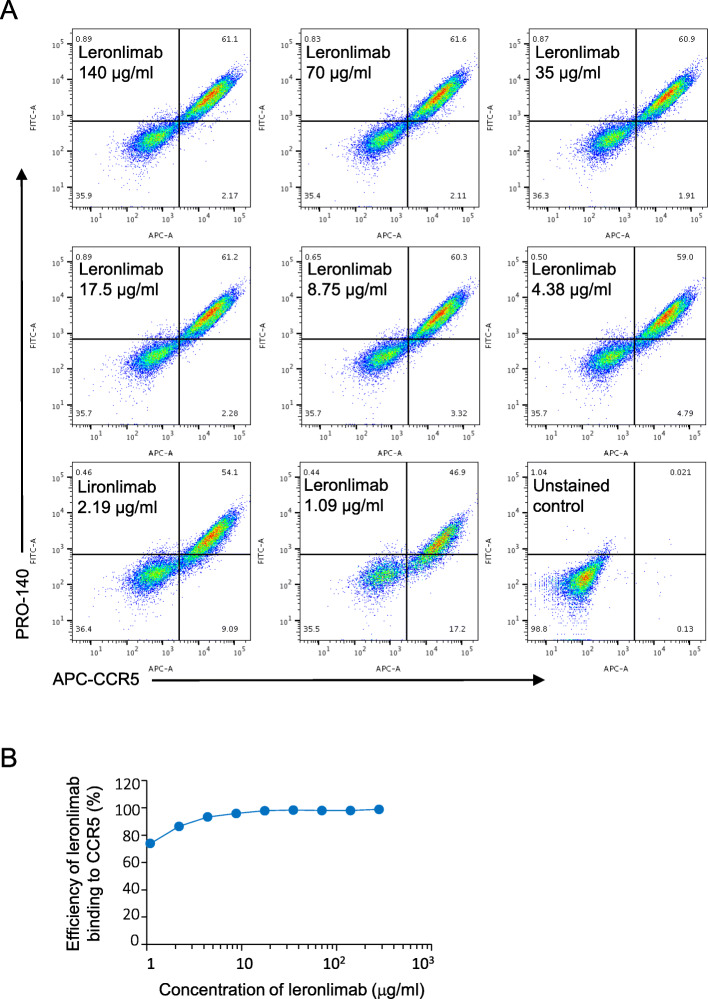
Leronlimab binds CCR5 in human breast cancer cells. **a** In order to determine the binding of leronlimab to human CCR5 in breast cancer cells, we used an MDA-MB-231 human breast cancer cell line transfected with a human CCR5 expression vector as a model system (MDA-MB-231-CCR5 cells). A commercial APC conjugated mouse anti-human/mouse/rat CCR5 antibody from R&D (FAB1802A) (APC-αCCR5), was used as a positive control to assess CCR5 positive cells. MDA-MB-231-CCR5 cells were stained with both APC-αCCR5 and leronlimab using the concentration from 1 to 140 mg/ml. Alexa Fluor 488-conjugated mouse anti-human IgG was used as a secondary antibody to measure leronlimab binding cells. Analysis of leronlimab binding with CCR5 by FACS is shown in **a**. Leronlimab binding with human CCR5 was validated. **b** The efficiency of leronlimab binding to CCR5-positive cells was up to 98% compared with CCR5 antibody (FAB1802A)

In order to determine the binding of leronlimab to human CCR5 in breast cancer cells, we used an MDA-MB-231 human breast cancer cell line transfected with a human CCR5 expression vector as a model system. A commercial APC-conjugated mouse anti-human/mouse/rat CCR5 antibody from R&D (FAB1802A) which we had previously tested was used as a positive control to assess CCR5-positive cells. MDA-MB-231-CCR5 cells were stained with leronlimab, and the concentration ranged from 1 to 140 μg/ml. Alexa Fluor 488-conjugated mouse anti-human IgG was used as a secondary antibody to measure leronlimab binding to cells. A commercially available APC-conjugated CCR5 antibody was used as a positive control for counterstaining. Analysis of leronlimab binding with CCR5 by FACS is shown in Fig. 1a. The efficiency of leronlimab binding to CCR5-positive cells was up to 98% (Fig. 1b).

The binding of leronlimab to endogenous CCR5 was next assessed by FACS comparing the binding of APC-labeled commercial CCR5 antibody (FAB1802A) with Alex fluor 488-labeled leronlimab in two human breast cancer cell lines (MD-MB-231 and SUM-159 (Supplemental Figure 1 (see Additional file 1)). The staining of cells with both antibodies was shown as a region of double-positive cells in the upper right quadrant for both cell lines (Supplemental Figure 1 (see Additional file 1)).

### Leronlimab blocks CCL5, CCL3, and CCL4 induced calcium signaling in breast cancer cells

Previous studies had shown that a subpopulation of MDA-MB-231 cells was positive for CCR5 [14]. CCR5 activation induces calcium flux [14]. To more effectively assess the effects of leronlimab on CCR5 function, we created a stable CCR5 transfected MDA-MB-231 cell line with a CCR5 expression vector.

**Fig. 2:**
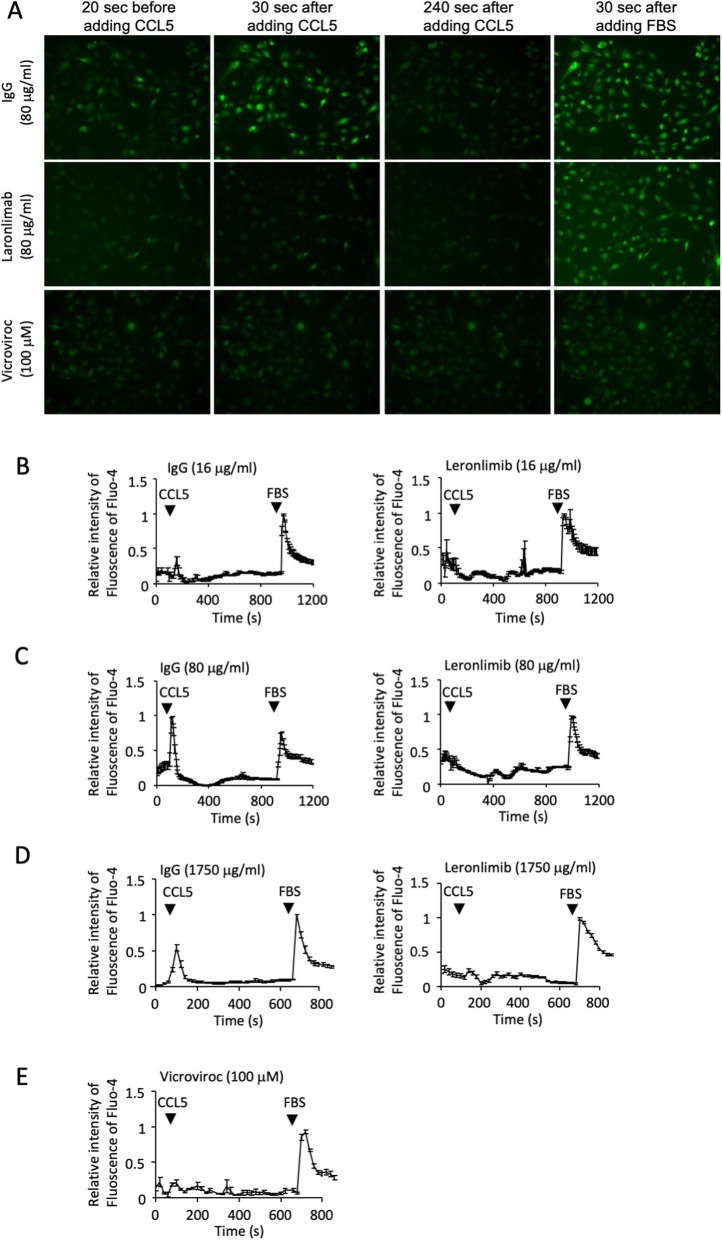
Leronlimab blocks human CCL5-CCR5-mediated signaling in human breast cancer cells. **a** MDA-MB-231-CCR5 cells were assessed for Ca^+ 2^ fluxes using Fluo-4 as a calcium concentration indicator. Fluorescence was measured at time points after the addition of the CCR5 ligand, CCL5, in the presence of either CCR5 inhibitors (leronlimab, vicriviroc) or control IgG. **b**–**e** Quantitation of the time course of Ca^+ 2^-induced fluorescence was obtained from living cell imaging (**a**–**d**). The CCR5 antagonist, vicriviroc, was used as a positive control (**a**, **e**). Leronlimab reduced CCL5-induced calcium responses in MDA-MB-231-CCR5 cells (1.23 ± 0.10, *N* = 10 for control cells and 0.54 ± 0.13 *N* = 12 for leronlimab-treated cells. *P* < 0.001 at calcium peak induce by CCL5)

**Fig. 3:**
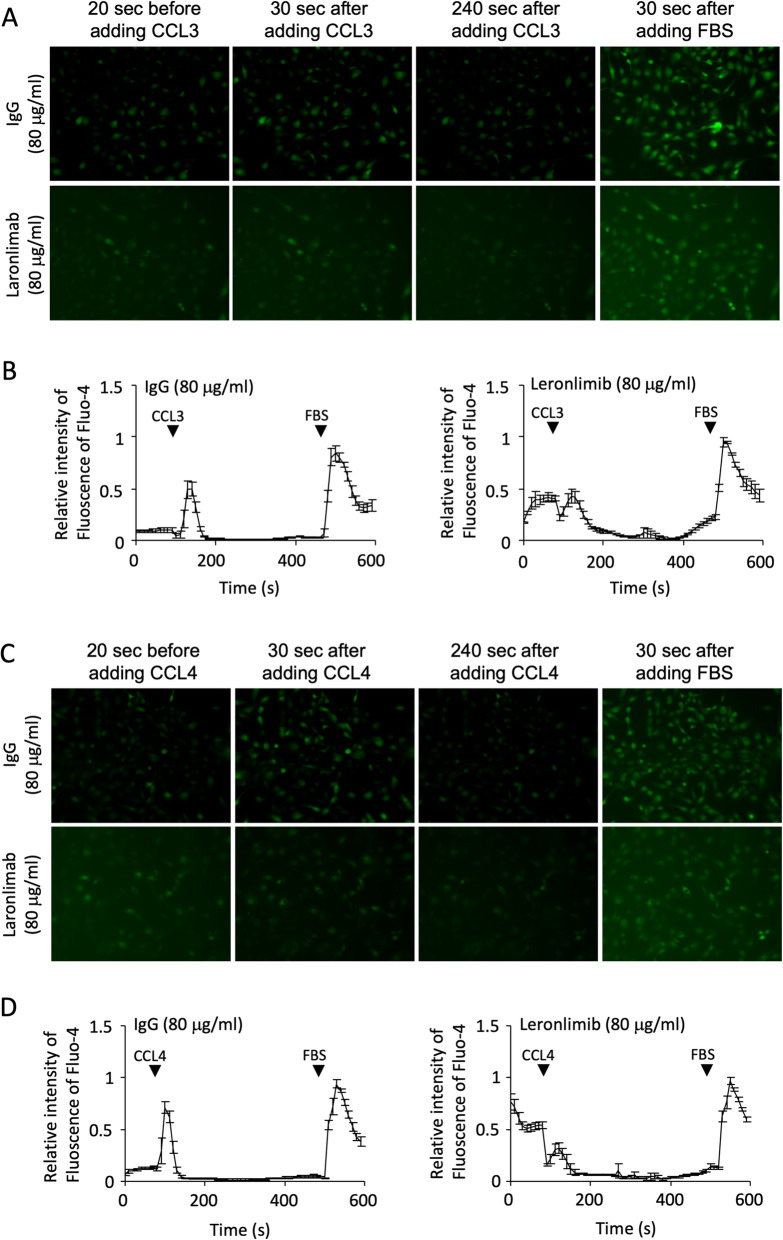
Leronlimab blocks human CCR5-mediated signaling by CCL3 and CCL4 in human breast cancer cells. **a** MDA-MB-231-CCR5 cells were assessed for Ca^+ 2^ fluxes using Fluo-4 as a calcium concentration indicator. Fluorescence was measured at time points after the addition of either CCL3 (**a**, **b**) or CCL4 (**c**, **d**) in the presence of either CCR5 inhibitors (leronlimab, vicriviroc) or control IgG. **b**, **d** Quantitation of the time course of Ca^+ 2^-induced fluorescence was obtained from living cell imaging (**a**, **c**)

Calcium responses were measured by fluorescent living cell imaging with Fluo-4 used as a calcium concentration indicator (Figs. 2 and 3). CCL5-induced calcium responses were shown in Fig. 2. Leronlimab concentration ranged from 80 to 1750 μg/ml (Fig. 2a–d). Human IgG was used as a negative control. Vicriviroc, a CCR5 antagonist, was used as a positive control (Fig. 2a, e). The results showed that leronlimab can block CCL5-induced calcium responses in MDA-MB-231-CCR5 cells with a concentration as low as 16 μg/ml (1.23 ± 0.10, *N* = 10 for control cells and 0.54 ± 0.13 *N* = 12 for leronlimab treated cells. *P* < 0.001 at calcium peak induce by CCL5).

Fluo-4 was used as a calcium concentration indicator to assess the impact of leronlimab on MDA-MB-231-CCR5 cells by living cell imaging (Fig. 2a–c). The CCR5 antagonist, vicriviroc, was used as a positive control (Fig. 2a, e). The results showed that leronlimab blocks CCL5-induced calcium responses in MDA-MB-231-CCR5 cells (1.23 ± 0.10, *N* = 10 for control cells and 0.54 ± 0.13, *N* = 12 for leronlimab-treated cells. *P* < 0.001 at calcium peak induced by CCL5.

### Leronlimab blocks human CCL3- and CCL4-induced Ca^+ 2^ responses in human breast cancer cells

Other ligands such as CCL3 and CCL4 can also bind to CCR5. Leronlimab abrogated CCL3 (Fig. 3a, b) and CCL4 (Fig. 3c, d) induced Ca^+ 2^ flux in MDA-MB-231-CCR5 cells (Fig. 3).

### Leronlimab blocks CCR5-mediated invasion of human breast cancer cells into the extracellular matrix

**Fig. 4:**
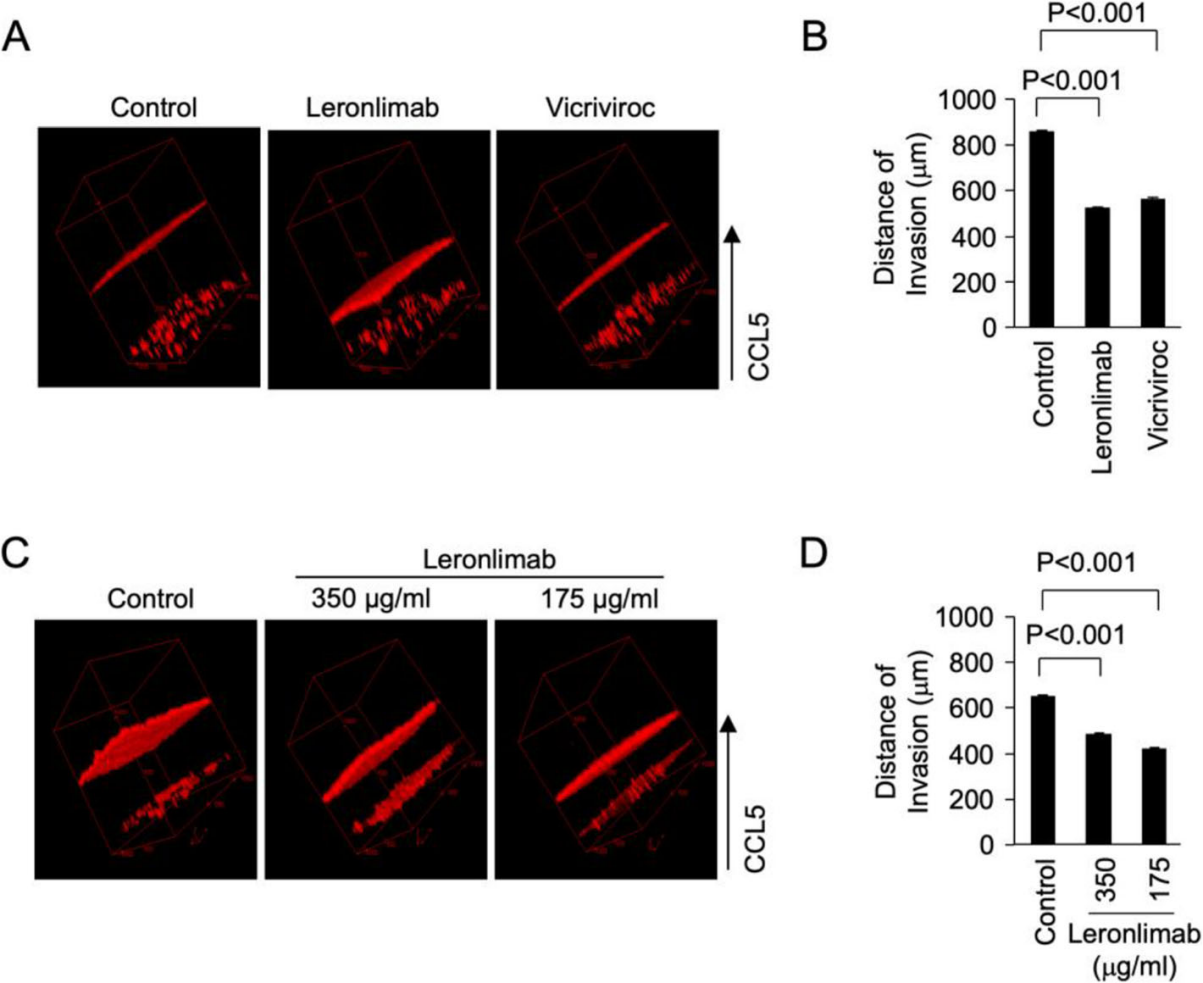
Leronlimab blocks CCR5-mediated invasion of human breast cancer cells into the extracellular matrix. To test the ability of leronlimab to block cell invasion in 3D Matrigel invasion assay, MDA-MB-231 cells were used. CCL5 was used as chemoattractant to induce invasion. The small-molecule inhibitor of CCR5, vicriviroc, was used as a form of positive control. Leronlimab reduced CCL5-induced MDA-MB-231 breast cancer cell invasion with similar efficacy as vicriviroc (**a**, **b**) (855 ± 8.7, *N* = 8 for control vs. 520 ± 9.1, *N* = 9 for leronlimab, *P* < 0.001). We also tested the effects of leronlimab doses on breast cancer cell invasion, and the results showed that both 175 and 350 μg/ml of leronlimab can effectively block MDA-MB-231 cell invasion (**c**, **d**)

The ability of breast cancer cells to invade extracellular matrix is distinguishable from but an important step in tumor metastasis. To test the ability of leronlimab to block cell invasion in 3D Matrigel invasion assay, MDA-MB-231 cells were used. CCL5 was used as a chemoattractant to induce invasion. The small-molecule inhibitor of CCR5, vicriviroc, was used as a form of positive control. Leronlimab reduced CCL5-induced MDA-MB-231 breast cancer cell invasion with similar efficacy as vicriviroc (Fig. 4a, b) (855 ± 8.7, *N* = 8 for control vs. 520 ± 9.1 μM distance traveled, *N* = 9 for leronlimab, *P* < 0.001). We also tested the effects of different doses of leronlimab on breast cancer cell invasion, and the results showed that both 175 and 350 mg/ml of leronlimab can effectively block MDA-MB-231 cell invasion (Fig. 4c, d). Thus, the pro-invasive effect of CCR5 can be abrogated by a humanized monoclonal antibody to CCR5.

### Leronlimab prevents breast cancer cell metastasis in a mouse lung metastasis model

**Fig. 5:**
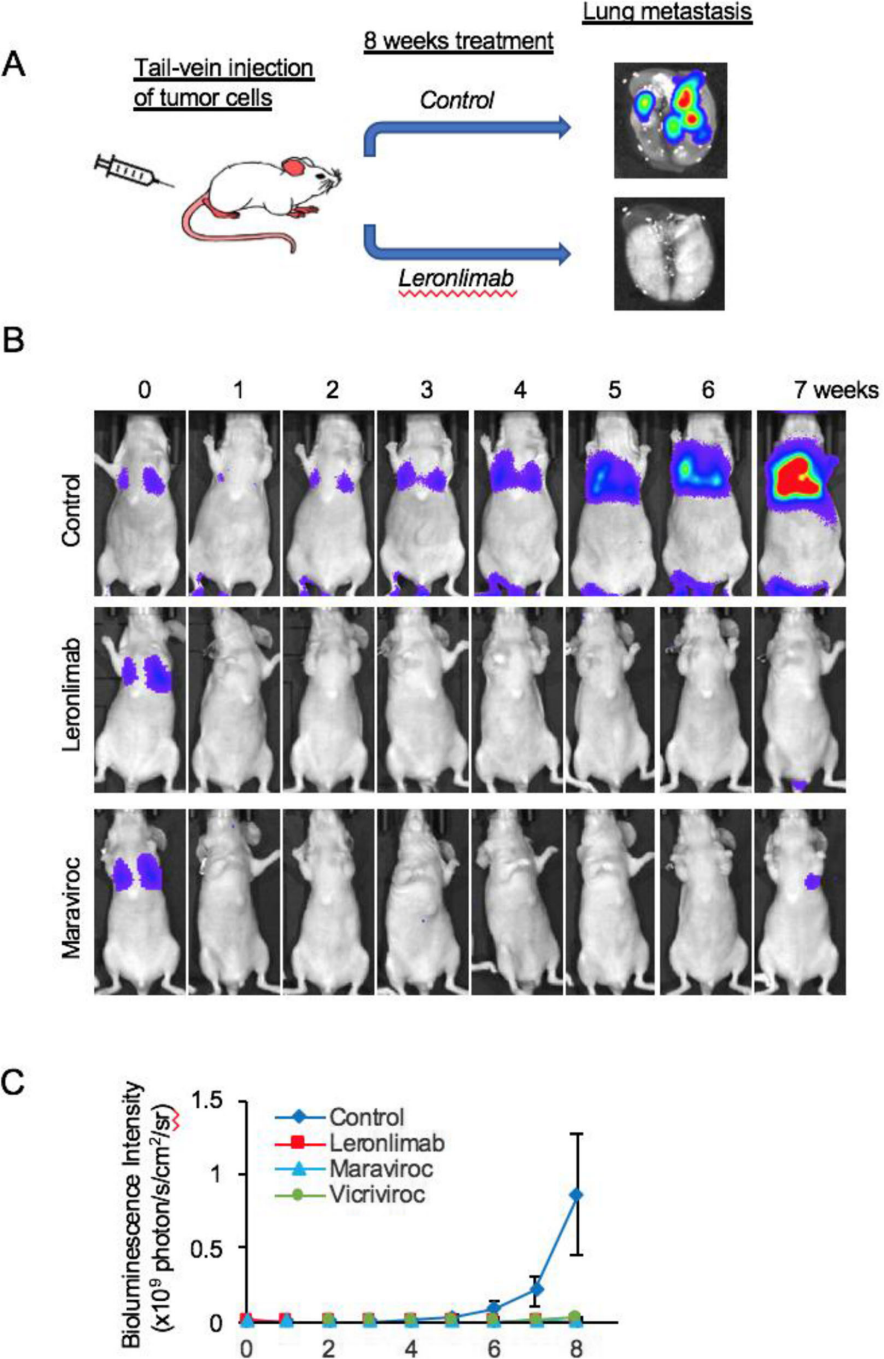
Leronlimab blocks breast cancer metastasis in mice (**a**). The mice were divided randomly into 4 groups (control, leronlimab, maraviroc, and vicriviroc). MDA-MB-231 cells stably transfected with Luc2-GFP were injected into the mice via the tail vein. The mice in each group were pretreated 1 day before injection with breast cancer cells. The volume of the metastatic tumor formed in the lung was determined by bioluminescence imaging. The bioluminescence images of representative mice from the control, leronlimab, and maraviroc groups are shown in **b**. The quantitative analysis of tumor size in each group is shown in **c**. The size of tumors is defined by photon flux (× 10^9^ p/s/cm^2^/sr). The data was show as mean ± SE. Leronlimab pretreatment decreased breast cancer tumor metastasis to the lung

Leronlimab blocks breast cancer metastasis in vivo. In view of the finding that CCR5 inhibition by leronlimab reduced calcium signaling and cell invasion, we determined the in vivo effect of leronlimab on the formation of lung metastasis. As a form of control, maraviroc was deployed as previously described. We used MDA-MB-231 cells transduced with the Luc2-eGFP lentiviral vector (MDA.pFULG cells) as an experimental metastasis model. The codons within the Luc2 gene in this vector have been optimized for the expression in mammalian cells, and therefore, mammalian cells expressing this reporter are 10 to 100 times brighter than the unmodified Luc gene. After injection of MDA. pFULG cells into the tail vein of mice, noninvasive BLI enabled the early detection of breast cancer metastasis. Weekly BLI was conducted for 8 weeks, and the radiance antemortem was used as a surrogate measurement of tumor burden. The dose of leronlimab was based on the bioequivalent dose shown to be safe in patients with HIV (700 mg) and the dose previously used to treat GvHD in mice [31]. Mice treated with leronlimab (2 mg/mouse) or maraviroc (8 mg/kg twice daily) showed a significant reduction in the volume of pulmonary metastases compared with vehicle-treated mice at 8 weeks (Fig. 5a, b, 860 × 10^6^ (*n* = 22 mice) vs. 3.7 × 10^6^ photons/s/cm^2^/sr (*n* = 6 mice) for leronlimab, vs. 0.4x × 10^6^ (*N* = 7) for maraviroc). Leronlimab reduced lung metastatic burden > 98% at 8 weeks (99.6%). Collectively, these results provide evidence that the CCR5 antagonist leronlimab reduces the formation of lung metastasis in a murine xenograft model.

In order to determine whether leronlimab functioned to reduce a component of the metastatic phenotype assessed by transwell migration, human breast cancer cells known to undergo transwell migration and in these studies shown to express CCR5 were analyzed. Leronlimab (80 μg/ml) reduced CCL5-induced breast cancer transwell migration (MDA-MB-231 and SUM149 cell lines) (Supplemental Figure 2 (see Additional file 1). Data are shown as mean ± SEM for *N* = 8).

### Leronlimab enhances cell killing by DNA damage-inducing chemotherapy agents used for breast cancer treatment

**Fig. 6:**
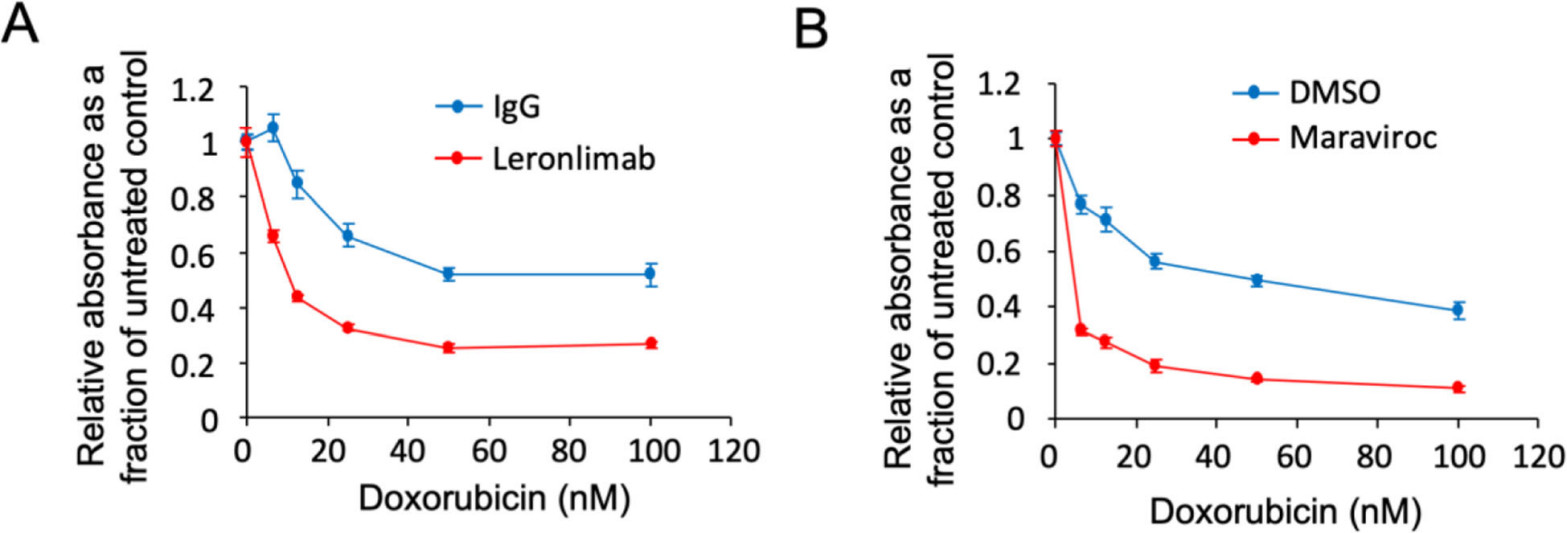
Leronlimab enhances the cell death induced by doxorubicin, a DNA damage-inducing chemotherapy agent. **a** MDA-MB-231 cells were treated with 10 μg/ml of leronlimab combined with different dose of doxorubicin for 3 days. The MTT assay was used to determine the relative cell number. The relative absorbance is shown as a fraction of the untreated control. The normalization of leronlimab-treated cells was to leronlimab with no doxorubicin. In **b**, the cells were treated with maraviroc (100 mM) combined with different doses of doxorubicin, used as a positive control. Data are shown as mean ± SEM for *N* = 8

Because CCR5 has been shown to activate DNA repair pathways [13], we investigated the potential for leronlimab to sensitize breast cancer cells to DNA-damaging agents. To test this hypothesis, we treated MDA-MB-231 cells with doxorubicin, a topoisomerase II inhibitor that induces DNA damage, together with either leronlimab (Fig. 6a) or maraviroc (Fig. 6b).

The addition of either leronlimab or maraviroc to doxorubicin decreased MTT activity, measured by OD570, reflecting reduced cell proliferation and cell death. Neither leronlimab nor maraviroc alone caused significant cytotoxicity (Fig. 6a, b). In order to determine whether the enhancement of doxorubicin mediated reduction in MTT activity was found in additional genetic types of breast cancer cell lines, we used FC-IBC-02, MDA-MB-436, and SUM149 (Supplemental Figure 3 (see Additional file 1)). Leronlimab enhanced the effect of doxorubicin to inhibit MTT activity in each cell line (*N* = 7, at 100 μm Doxorubicin, *P* < 0.001).

### Leronlimab reduces the volume of established progressing human breast cancer metastasis in mice

Our prior studies had demonstrated that leronlimab reduced the onset of breast cancer tumor metastasis to the lungs. We next conducted experiments in order to determine whether leronlimab could reduce the volume of established breast cancer metastasis. In order to determine the impact of leronlimab on established metastasis, we conducted an analysis of mice with lung metastasis from MDA-MB-231 cells.

**Fig. 7:**
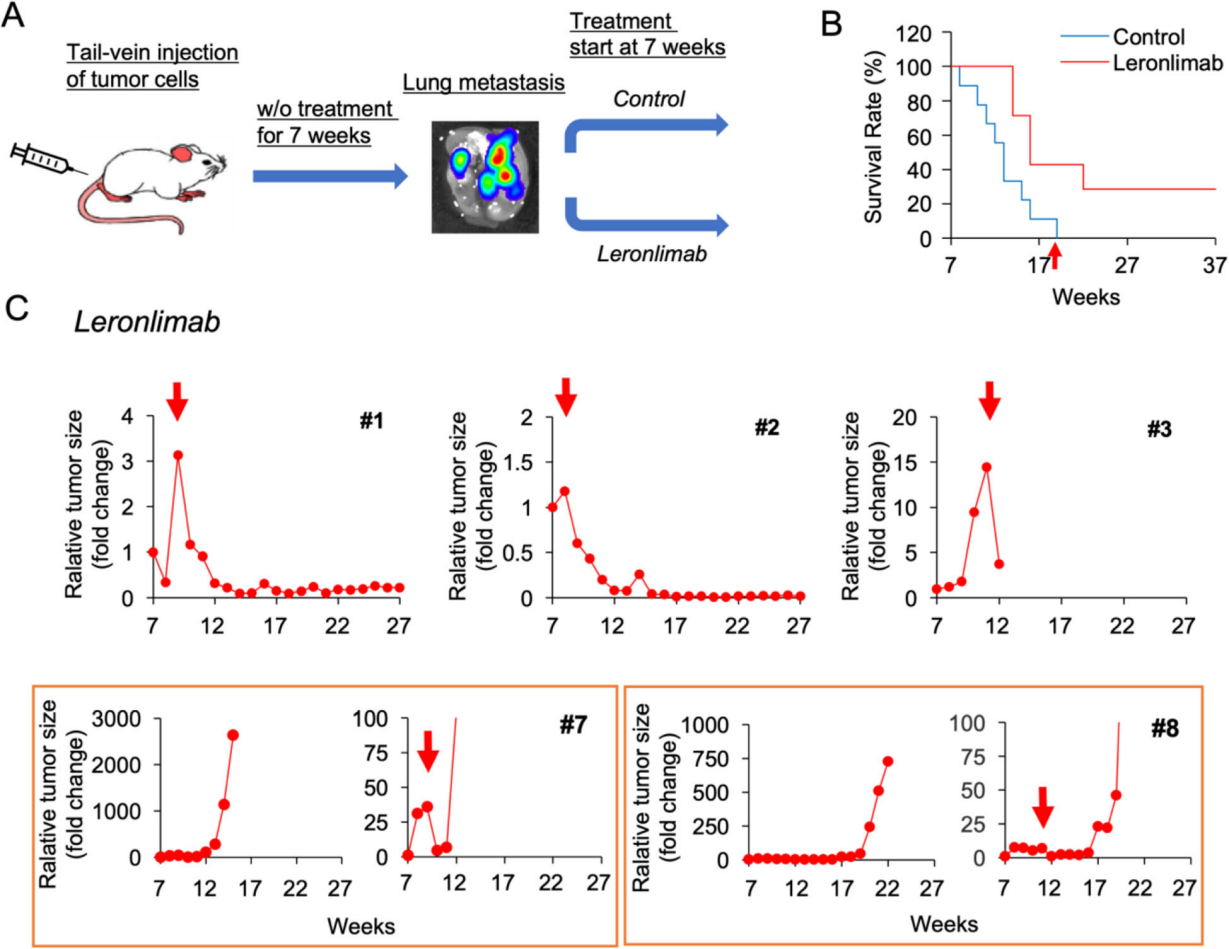
Leronlimab significantly increases survival in mice with established breast cancer lung metastasis. **a** Schematic representation of the study design. The mice were injected with MDA-MB-231-pFULG cells via the tail vein. After 7 weeks, when breast cancer lung metastasis was established, the mice were randomly assigned into two cohorts. One cohort was treated with leronlimab (2 mg/mouse, twice a week, 8 mice), and the other was as untreated control. **b** The survival of mice is plotted with time after the addition of treatment from week 7. **c** Representative examples of the tumor volume for mice treated with leronlimab, plotted with time after the addition of treatment from week 7. In five mice, the tumor volume decreased as shown (#1, #2, #3, #7, and #8. Mouse #3 was dead between 13 and 14 weeks due to fighting). The decrease in tumor size in mouse #1 was 3-fold to 0.1-fold; #2, 1.2-fold to 0.01-fold; #3, 14.4-fold to 3.7-fold; #7, 7-fold to 0.9-fold; and #8 35.9-fold to 4.7-fold)

Animals were injected with MDA.-pFULG cells into the tail vein of mice, and noninvasive weekly BLI was conducted for 7 weeks. The radiance antemortem was used as a surrogate measurement of tumor burden. After 7 weeks, when breast cancer lung metastasis was established, the tumor-bearing mice were divided randomly into two groups. Mice were then treated with leronlimab (2 mg/mouse, twice a week, 8 mice/group), and untreated mice were used as control (Fig. 7a). Prior to 7 weeks, all the animals had shown a progressive increase in metastatic tumor burden. For the purpose of the study, the tumor volume was determined for each mouse and normalized to 100% as the starting point of the intervention at 7 weeks, and tumor volume was followed weekly for each animal. In the control group, mouse death was documented from the first week, and all mice were dead by 19 weeks (Fig. 7b). In contrast, in the leronlimab-treated group, 42.9% were alive at 21 weeks and 28.6% remained alive at 37 weeks. Leronlimab treatment resulted in a significant reduction in survival rate (log-rank test, *p* = 0.021). None of the untreated animals showed a reduction in metastatic tumor burden (Supplemental Figure 4A,B (see Additional file 1)). In 5 out of 8 mice treated with leronlimab, a significant reduction in tumor metastatic volume was observed after 8 weeks (Fig. 7c) (Supplemental Figure 4C (see Additional file 1)).

We next conducted a histological analysis of the lung metastases from the mice post-mortem. In order to determine the relative area of the lung occupied by metastasis at death in the mice that were either treated with leronlimab or unreated, the mice were euthanized and the lungs analyzed after paraffin embedding. Longitudinal sections (4 μm) of the entire lung were obtained every 100 μm and stained with hematoxylin and eosin. Each section was evaluated to identify lesions and to differentiate lesions from other space-occupying alterations including consolidation and inflammation. The region of lung metastasis for each animal was quantified in a blinded fashion using Fiji ImageJ, and the mean data were compared as mean ± SEM for *N* = 5 separate mice (Supplemental Figure 5 (see Additional file 1)). These studies showed that the mean tumor size was significantly reduced in the leronlimab-treated mice.

## Discussion

CCR5 abundance is induced by transformation of immortalized human breast cells with diverse oncogenes including Ha-Ras, c-Myc, ErbB2, and c-Src [12]. Furthermore, DNA damage induced by radiation and chemotherapy is associated with increased expression of CCR5 within human breast cancer cell lines [13]. Increased cytoplasmic CCR5 abundance correlates with poor prognosis in a variety of cancers including breast cancer [13], gastric adenocarcinoma, and other malignancies [18]. The rationale for the current studies includes evidence that CCR5 may participate in the metastatic progression of breast cancer [12]. In the current studies, we show that the humanized monoclonal antibody leronlimab efficiently blocks ligand-induced Ca^2^^+^ signaling, cellular invasion, and tumor metastasis. Prior findings had shown that CCR5 small-molecule antagonists (maraviroc and vicriviroc) block metastasis of human breast cancer xenografts (MDA-MB-231 cells) [12, 13]. The current studies extend these findings by demonstrating the humanized monoclonal antibody to CCR5, leronlimab, efficiently bound CCR5 expressed on human breast cancer cells, blocked ligand-induced Ca^2^^+^ signaling, and inhibited Matrigel invasion of breast cancer cells. Furthermore, leronlimab reduced tumor metastasis in immune-deficient mice. In a subset of mice with established TNBC lung metastasis, leronlimab reduced the metastatic tumor burden and increased overall survival. As leronlimab has been well tolerated in the HIV patient population without significant drug-related adverse events [18], the current studies suggest leronlimab may have clinical application.

The current studies showed that leronlimab blocks CCR5 signaling, assessed by calcium release in response to several distinct ligands. Upon binding of the ligand, the conformational change in CCR5 dissociates the Gαi and the Gβγ subunits, inducing downstream signaling. The activation of Ca^2^^+^ signaling and cellular migration by CCR5 is preserved in both immune cells [23] and cancer cells [12, 13]. CCR5 binds many ligands which are overexpressed in the tumor microenvironment including CXCL13 (BCA-1), CCL3 (MIP1α), CCL3L1, CCL4 (MP-1β), CCL8 (MCP2), CCL11 (eotaxin), CCL13 (MCP-4), and CCL16 (HCC-4). Elevated levels of CCL5 indicate poor prognosis in breast cancer [33, 34], pancreatic cancer [35], cervical cancer [34], prostate cancer, ovarian cancer [36], and gastric cancer [14, 37].

Herein, leronlimab enhanced cell killing by the DNA damaging agent doxorubicin. It is likely that two previously described mechanisms contribute to these findings. Firstly, CCR5 induces both homologous and non-homologous DNA repair [13]. Secondly, cell survival signaling pathways, ribosomal biogenesis, and PI3K/Akt are induced by CCR5 when analyzed by single-cell analysis of breast cancer cells. Many transcripts were induced (> 1000-fold) by the expression of CCR5 when compared to neighboring CCR5^−^ breast cancer cells [13]. The induction of the PI3K pathway and thereby PDK1 and serine/threonine kinase protein kinase B (AKT) pathway, by CCR5, in turn, induces cell survival, glycolysis, cell proliferation, growth and proliferation of progenitor and stem cells, immune cell differentiation, and the release of eIF4E to promote cap-dependent translation [12, 13].

A substantial number of studies have provided evidence in other systems that CCR5 participates in the important anti-tumor immune response. In the current studies, leronlimab restrained the development of tumor metastasis in murine xenografts in Nu/Nu mice which lack functional T cells [38]. The nude mouse (nu or Hfh11nu or Foxn1nu) lack a thymus due to a mutation in the *FOXN1* gene. The absence of a thymus means that there is no production of T cells; therefore, they are unable to activate the different types of immune responses (adaptive) during the implantation of cancer cells. These mice lack antibody formation, cell-mediated immune responses, and delayed-type hypersensitivity responses but produce NK cells [39], resulting in a reduced capability of killing virus-infected or malignant cells [38]. Our studies suggest therefore that T cell participation is not necessary for the anti-tumor function of leronlimab observed in the current studies but do not exclude a potential role for NK cells which express CCR5 [18]. Furthermore, as leronlimab is a humanized antibody that does not bind murine cells, it is most likely the effect seen with leronlimab is mediated directly on the human breast cancer cells, rather the local murine tumor environment. That said, evidence supports a model in which additional immune functions are regulated by CCR5 and T cells in other settings. CCL5 recruits CCR5-expressing TAMs [40, 41]. T cells participate in the anti-tumor immune responses, in part through CCR5-dependent regulation of macrophage differentiation [42]. The recruitment of immune cells, including tumor-infiltrating lymphocytes (TILs), MDSCs, tumor-associated macrophages (TAMs), innate lymphoid cells (ILCs), Tregs [43], mesenchymal stem cells (MSCs), and immature dendritic cells (DCs), contributes to tumor-induced immunosuppression [44]. Many of these cell types express CCR5 and/or produce ligands for CCR5 [18]. Prior studies showed the small molecule CCR5 inhibitor maraviroc reduced MDSC-induced colon cancer metastasis [45]. In the phase 1 pilot MARACON study, patients with advanced-stage metastatic colorectal cancer that were refractory to current therapies [46] were treated with maraviroc. CCR5 inhibition correlated with reduced proliferation and an anti-tumoral macrophage polarized M1 morphology [46], although more complex interactions occur with PD-1- and CTLA-4-positive cells surrounding tumors with patchy CCR5 expression [47].

The current studies extend prior studies demonstrating the importance of CCR5 in breast tumor metastasis prevention and by showing for the first time a reduction in the volume of established metastasis with life extension. The requirement for CCR5 in oncogene-induced cellular proliferation was supported by transgenic studies in which MMTV-PyMT-induced mammary tumors were reduced in *CCR5*^*−/−*^ mice [48]. Multiple CCR5-mediated pathways may contribute to tumor progression including MDSC [49], vascularity, and lymphangiogenesis [50, 51]. CCR5 siRNA did not reduce the metastatic phenotype of MDA-MB-231 cells in the absence of additional MDSC [52], endothelial cells produce CCL5, and augmented breast cancer metastasis in another study [49]. In addition, CCR5 inhibitors also reduced lymphangiogenesis in triple-negative breast cancer (TNBC) cell line xenografts [50, 51]. Other approaches to restrain tumor metastasis via CCR5 inhibition include targeting CCL5 in the bone marrow via nanoparticle-delivered expression silencing, in combination with maraviroc, which augmented anti-tumor immunity [53].

It is likely that CCR5 plays a broader role in governing cancer metastasis as maraviroc and vicriviroc reduced prostate cancer cell metastasis to the bones, brain, and viscera in immune-competent mice [22] and reduced metastasis or cellular migration in glioblastoma [54] and a variety of other malignancies [18]. Prior studies had shown that CCR5 induces cancer cell homing to metastatic sites [12, 55], augments the pro-inflammatory pro-metastatic immune phenotype [46], and enhances DNA repair [13], providing aberrant cell survival and resistance to DNA-damaging agents. The current studies, showing a reduction in the volume of established breast cancer metastasis with life extension, provide support for a controlled clinical intervention study using leronlimab in patients with TNBC.

## Conclusion

Our studies show that the humanized monoclonal antibody, leronlimab, directed to the G protein-coupled receptor, CCR5, can both prevent breast cancer metastasis and reduce established metastasis. As CCR5 is expressed on the surface of breast cancer cells and leronlimab reduced CCR5-dependent cell-autonomous functions, including calcium signaling and cellular invasion, the impact of leronlimab in this case is likely mediated via a direct effect on the breast cancer cells. The studies were conducted in immune-deficient Nu/Nu mice, suggesting certain immune functions are not necessary for the action of leronlimab on TNBC metastasis in vivo. Leronlimab is administered as a weekly subcutaneous injection and has been used in more than 800 patients with HIV, without serious adverse events related to the drug. Together these findings suggest additional clinical studies of leronlimab in metastatic human breast cancer are warranted.

## Supplementary Information

**Additional file 1: Supplemental Figures. Supplemental Figure 1.** Leronlimab binds endogenous CCR5. Breast cancer cell line MDA-MB-231 (A) and SUM-159 (B) were assessed by FACS comparing the binding of APC-labelled commercial CCR5 antibody (FAB1802A) with FITC-labelled leronlimab. The staining of cells with both antibodies was shown as a region of double positive cells in the upper right quadrant. **Supplemental Figure 2.** Leronlimab blocks CCR5-mediated invasion of genetically distinct breast cancer cell lines into extracellular matrix. To test the ability of leronlimab to block transwell migration, CCL5 was used as chemoattractant to induce migration. Leronlimab reduced CCL5-induced breast cancer transwell migration **(**MDA-MB-231, SUM159 cell lines). Data are shown as mean ± SEM for *N*= 6. **Supplemental Figure 3.** Leronlimab enhances the cell death induced by Doxorubicin in multiple distinct breast cancer cell lines. MDA-MB-231 cells was treated with 10 μg/ml of leronlimab combining with a 50 or 100 nM dose of doxorubicin for 3 days. The methylene blue staining (for FC-IBC-02 and MDA-MB-436 cells, read at 650 nm) or MTT (for SUM149 cell, read at 570 nm) assay were used to determine the relative cell number. Comparison was made with equimolar amounts of control human IgG. Data are shown as mean ± SEM for *N*= 7. **Supplemental Figure 4.** Leronlimab reduces the size of established breast cancer lung-metastasis. (A). Schematic representation of study design. The mice were injected with MDA-MB-231-pFULG cells via the tail-vein. After 7 weeks, when breast cancer lung metastasis was established, the mice were randomly assigned into two cohorts. One cohort was treated with leronlimab (2 mg/mouse, twice a week, 8 mice/group) and the other with control. (B). Representative examples of the metastatic tumor volume for mice treated with control or (C). leronlimab, plotted with time after the addition of treatment from week 7. In five mice, the tumor volume decreased as shown (#1, #2, #3, #7 and #8). **Supplemental Figure 5.** Leronlimab significantly reduces breast cancer lung-metastasis. (A). Representative H&E staining of lung sections from mice either treated with control or leronlimab (2mg/kg) by the protocol shown in **Supplemental Figure 4.** Lungs were removed post-mortem and the relative area of the lung occupied by metastasis quantified for the leronlimab vs. control treated groups (B). Data (mean ± SEM) are shown for the ratio of tumor area vs. whole lung area (*N*= 5 separate mice/group). Dotted lines in (A) delineate the circumference of the tumor area.

## Data Availability

All data generated or analyzed during this study are included in this published article and its supplementary information files.
