# Supplementary material for: Leronlimab, a humanized monoclonal antibody to CCR5, blocks breast cancer cellular metastasis and enhances cell death induced by DNA damaging chemotherapy
Source: Breast Cancer Res. 2021 Jan 23;23:11. doi: 10.1186/s13058-021-01391-1 (PMC7825185; doi:10.1186/s13058-021-01391-1)
Supplement: Supplementary file 1 — Additional file 1: Supplemental Figures. Supplemental Figure 1. Leronlimab binds endogenous CCR5. Breast cancer cell line MDA-MB-231 (A) and SUM-159 (B) were assessed by FACS comparing the binding of APC-labelled commercial CCR5 antibody (FAB1802A) with FITC-labelled leronlimab. The staining of cells with both antibodies was shown as a region of double positive cells in the upper right quadrant. Supplemental Figure 2. Leronlimab blocks CCR5-mediated invasion of genetically distinct breast cancer cell lines into extracellular matrix. To test the ability of leronlimab to block transwell migration, CCL5 was used as chemoattractant to induce migration. Leronlimab reduced CCL5-induced breast cancer transwell migration (MDA-MB-231, SUM159 cell lines). Data are shown as mean ± SEM for N= 6. Supplemental Figure 3. Leronlimab enhances the cell death induced by Doxorubicin in multiple distinct breast cancer cell lines. MDA-MB-231 cells was treated with 10 μg/ml of leronlimab combining with a 50 or 100 nM dose of doxorubicin for 3 days. The methylene blue staining (for FC-IBC-02 and MDA-MB-436 cells, read at 650 nm) or MTT (for SUM149 cell, read at 570 nm) assay were used to determine the relative cell number. Comparison was made with equimolar amounts of control human IgG. Data are shown as mean ± SEM for N= 7. Supplemental Figure 4. Leronlimab reduces the size of established breast cancer lung-metastasis. (A). Schematic representation of study design. The mice were injected with MDA-MB-231-pFULG cells via the tail-vein. After 7 weeks, when breast cancer lung metastasis was established, the mice were randomly assigned into two cohorts. One cohort was treated with leronlimab (2 mg/mouse, twice a week, 8 mice/group) and the other with control. (B). Representative examples of the metastatic tumor volume for mice treated with control or (C). leronlimab, plotted with time after the addition of treatment from week 7. In five mice, the tumor volume decreased as shown (#1, #2, #3, #7 [file 13058_2021_1391_MOESM1_ESM.pdf]

Leronlimab, a humanized monoclonal antibody to CCR5, blocks breast cancer cellular metastasis and enhances cell death induced by DNA damaging chemotherapies.

Xuanmao Jiao[1*], Min Wang[1], Zhao Zhang[1], Zhiping Li[1], Dong Ni[1], Anthony W. Ashton[1,4], Hsin-Yao Tang[2], David W. Speicher[2], Richard G. Pestell[1,2,3*]

SUPPLEMENTAL FIGURES

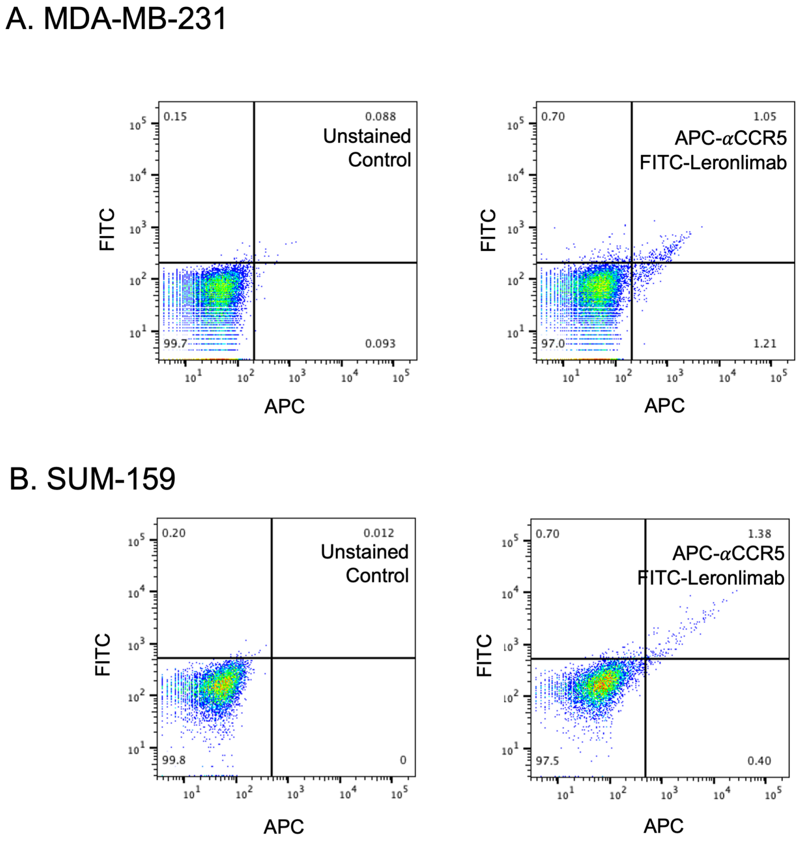

Supplemental Figure 1. Leronlimab binds endogenous CCR5. Breast cancer cell line MDA-MB-231 (A) and SUM-159 (B) were assessed by FACS comparing the binding of APC-labelled commercial CCR5 antibody (FAB1802A) with FITC-labelled leronlimab. The staining of cells with both antibodies was shown as a region of double positive cells in the upper right quadrant.

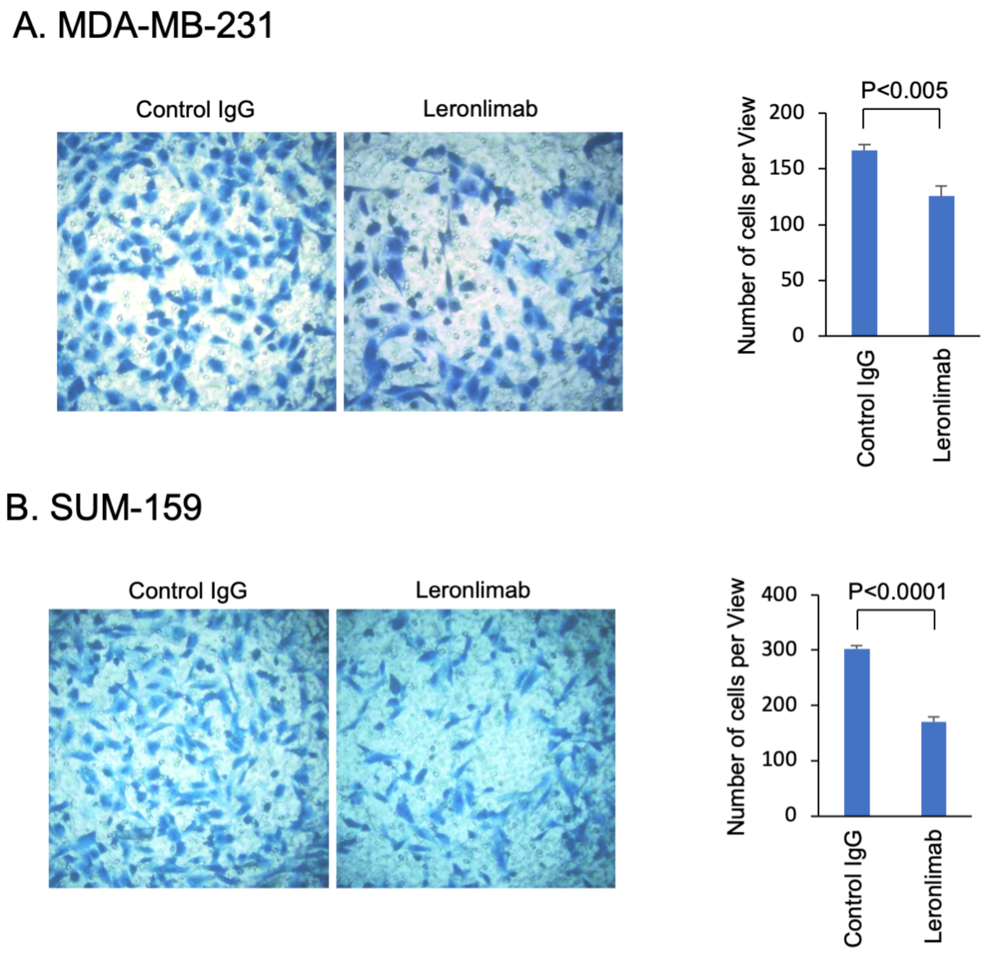

Supplemental Figure 2. Leronlimab blocks CCR5-mediated invasion of genetically distinct breast cancer cell lines into extracellular matrix. To test the ability of leronlimab to block transwell migration, CCL5 was used as chemoattractant to induce migration. Leronlimab reduced CCL5-induced breast cancer transwell migration (MDA-MB-231, SUM159 cell lines). Data are shown as mean ± SEM for N= 6.

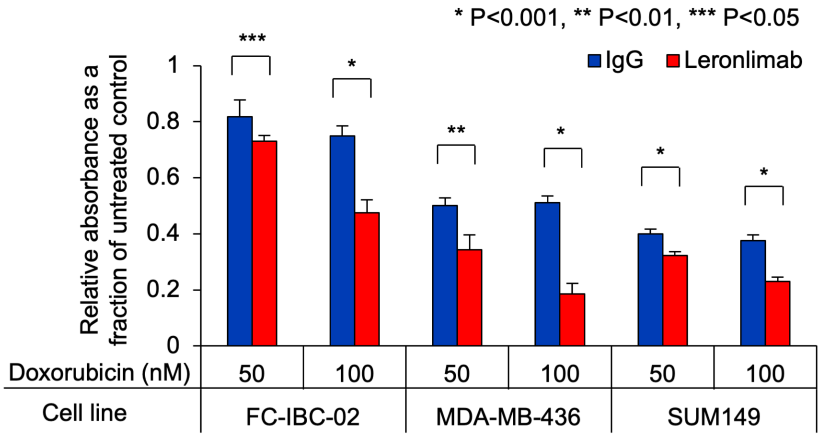

Supplemental Figure 3. Leronlimab enhances the cell death induced by Doxorubicin in multiple distinct breast cancer cell lines. MDA-MB-231 cells was treated with 10 µg/ml of leronlimab combining with a 50 or 100 nM dose of doxorubicin for 3 days. The methylene blue staining (for FC-IBC-02 and MDA-MB-436 cells, read at 650 nm) or MTT (for SUM149 cell, read at 570 nm) assay were used to determine the relative cell number. The relative absorbance is shown as a fraction of the untreated control. The normalization of leronlimab treated cells was to leronlimab with no doxorubicin. Comparison was made with equimolar amounts of control human IgG. Data are shown as mean ± SEM for N= 7.

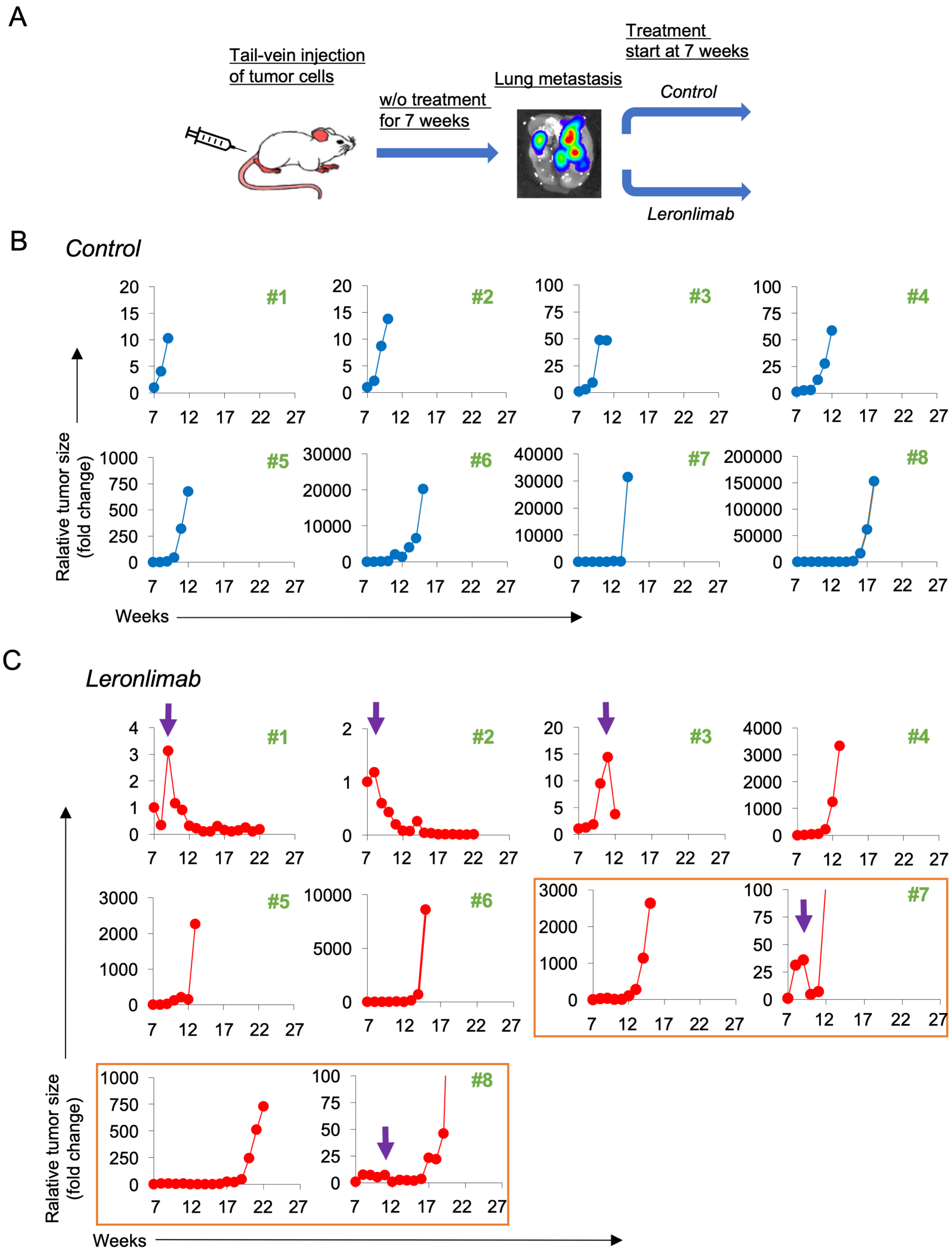
A
Tail-vein injection of tumor cells
w/o treatment for 7 weeks
Lung metastasis
Treatment start at 7 weeks
Control
Leronlimab
B
Control
Ralative tumor size (fold change)
Weeks
#1
#2
#3
#4
#5
#6
#7
#8
C
Leronlimab
Ralative tumor size (fold change)
Weeks
#1
#2
#3
#4
#5
#6
#7
#8

Supplemental Figure 4. Leronlimab reduces the size of established breast cancer lung-metastasis. (A). Schematic representation of study design. The mice were injected with MDA-MB-231-pFULG cells via the tail-vein. After 7 weeks, when breast cancer lung metastasis was established, the mice were randomly assigned into two cohorts. One cohort was treated with leronlimab (2 mg/mouse, twice a week, 8 mice/group) and the other with control. (B). Representative examples of the metastatic tumor volume for mice treated with control or (C). leronlimab, plotted with time after the addition of treatment from week 7. In five mice, the tumor volume decreased as shown (#1, #2, #3, #7 and #8).

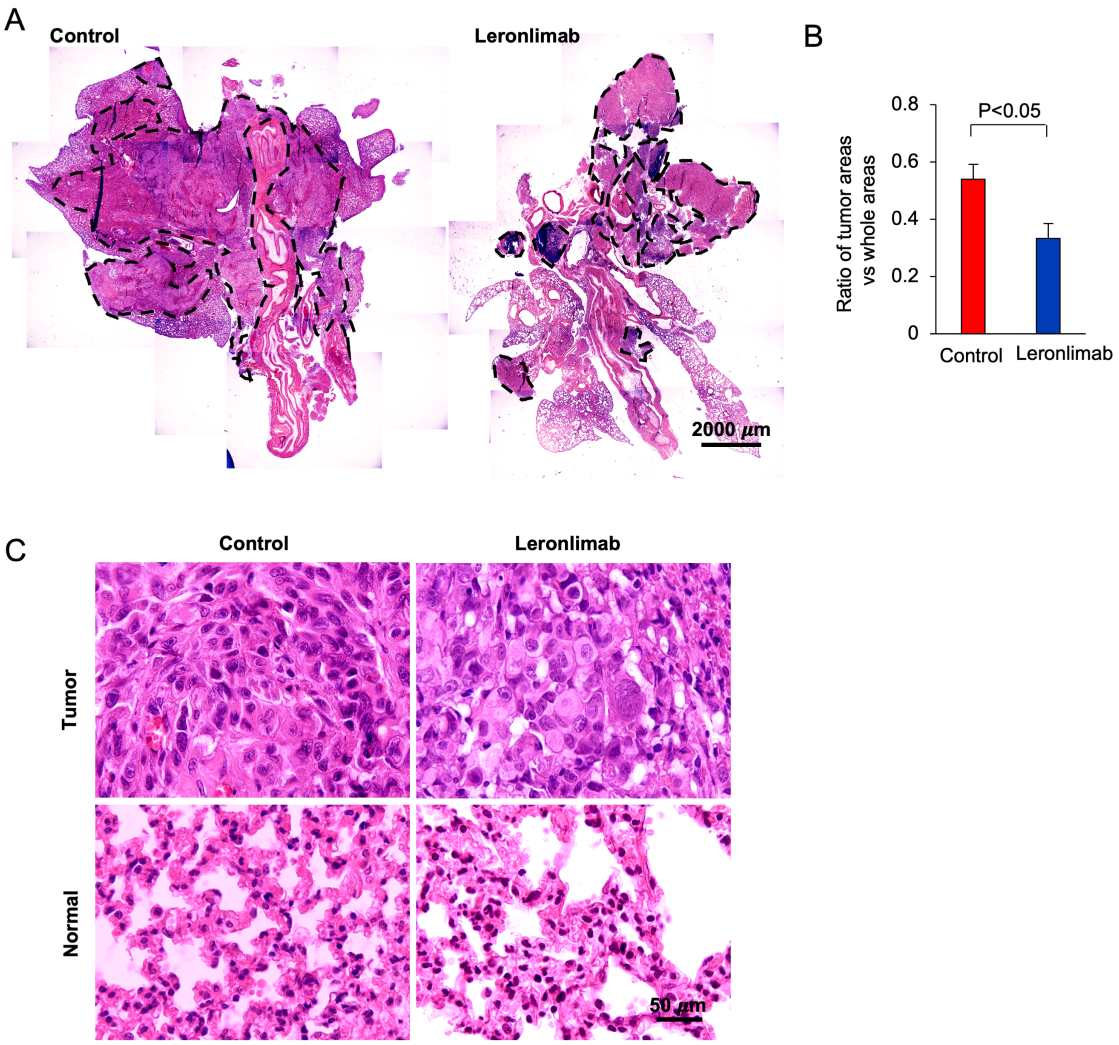

Supplemental Figure 5. Leronlimab significantly reduces breast cancer lung-metastasis. (A). Representative H&E staining of lung sections from mice either treated with control or leronlimab (2mg/kg) by the protocol shown in **Supplemental Figure 4.** Lungs were removed post-mortem and the relative area of the lung occupied by metastasis quantified for the leronlimab *vs.* control treated groups (B). Data (mean ± SEM) are shown for the ratio of tumor area *vs.* whole lung area (N= 5 separate mice/group). Dotted lines in (A) delineate the circumference of the tumor area. (C). Representative hi-resolution images taken from both normal and tumor areas in control or leronlimab treated mice.
